# Methodology and application of *Escherichia coli* F4 and F18 encoding infection models in post-weaning pigs

**DOI:** 10.1186/s40104-019-0352-7

**Published:** 2019-06-13

**Authors:** Diana Luise, Charlotte Lauridsen, Paolo Bosi, Paolo Trevisi

**Affiliations:** 1 Department of Agricultural and Food Sciences (DISTAL), Alma Mater Studiorum - University of Bologna, Bologna, Italy; 2 Faculty of Science and Technology, Department of Animal Science, Aarhus University, Tjele, Denmark

**Keywords:** Biomarkers, Challenge, ETEC, Piglet, Post-weaning

## Abstract

The enterotoxigenic *Escherichia coli* (ETEC) expressing F4 and F18 fimbriae are the two main pathogens associated with post-weaning diarrhea (PWD) in piglets. The growing global concern regarding antimicrobial resistance (AMR) has encouraged research into the development of nutritional and feeding strategies as well as vaccination protocols in order to counteract the PWD due to ETEC. A valid approach to researching effective strategies is to implement piglet *in vivo* challenge models with ETEC infection. Thus, the proper application and standardization of ETEC F4 and F18 challenge models represent an urgent priority. The current review provides an overview regarding the current piglet ETEC F4 and F18 challenge models; it highlights the key points for setting the challenge protocols and the most important indicators which should be included in research studies to verify the effectiveness of the ETEC challenge.

Based on the current review, it is recommended that the setting of the model correctly assesses the choice and preconditioning of pigs, and the timing and dosage of the ETEC inoculation. Furthermore, the evaluation of the ETEC challenge response should include both clinical parameters (such as the occurrence of diarrhea, rectal temperature and bacterial fecal shedding) and biomarkers for the specific expression of ETEC F4/F18 (such as antibody production, specific F4/F18 immunoglobulins (Igs), ETEC F4/F18 fecal enumeration and analysis of the F4/F18 receptors expression in the intestinal brush borders). On the basis of the review, the piglets’ response upon F4 or F18 inoculation differed in terms of the timing and intensity of the diarrhea development, on ETEC fecal shedding and in the piglets’ immunological antibody response. This information was considered to be relevant to correctly define the experimental protocol, the data recording and the sample collections. Appropriate challenge settings and evaluation of the response parameters will allow future research studies to comply with the replacement, reduction and refinement (3R) approach, and to be able to evaluate the efficiency of a given feeding, nutritional or vaccination intervention in order to combat ETEC infection.

## Introduction

Post-weaning diarrhea (PWD) appears primarily during the first 2 weeks post-weaning of the piglet. According to the literature, the most diffuse etiological agents responsible for PWD in piglets are enterotoxigenic *Escherichia coli* (ETEC) displaying the fimbriae F4 and F18. To control the risk related to the occurrence of PWD, the improper use of antibiotic treatment during the first 2 weeks post-weaning is prevalent in pig production. As an alternative to treatment with antimicrobials, the administration of the supranutritional level of zinc oxide (ZnO) at 2500–3000 ppm is a common strategy; however, this strategy has been banned by the European Union (EU) Commission beginning in 2022 [1]. The increased awareness of the use of antibiotics and ZnO is due to the growing risk of the occurrence of antimicrobial resistance (AMR) and of their environmental impact. In Europe, a recent limitation regarding the use of antibiotics, even for therapeutic purposes (e.g., colistin), has arrived. Hence, there is an increased and emergent interest in developing new strategies to limit the occurrence of PWD in pig production, and scientists, veterinarians, and nutritionists are trying to identify solutions for preventing and treating PWD. However, this is a major challenge and, according to the authors’ knowledge, no ‘silver bullet’ has yet been identified to cope with PWD. Previous reviews have described nutritional and feeding strategies, such as supplementation with organic and inorganic acids [2], essential oils and herbs [3], and some types of probiotics, prebiotics and symbiotics [4], different dosages of essential amino acids [5] and nucleotides [6, 7], or the potential use of bacteriophages [8] to prevent and counteract PWD. In order to research effective strategies with the potential of counteracting PWD, a valid approach is to implement *in vivo* challenge models with ETEC infection.

The most diffuse *in vivo* challenge models are based on lipopolysaccharide (LPS); ETEC or ETEC twinned with circovirus. LPS is the outer surface of all Gram-negative bacteria; it causes acute immune stimulation by means of the activation of several signalling pathways, (e.g., TLR4 and CD14) resulting in a cascade of syntheses of cytokines, miming many aspects of the inflammatory process of pathogens [9, 10]. However, the *in vivo* challenge model with LPS poses some concerns including 1) the development of endotoxin tolerance by the host, defined as reduced responsiveness to the LPS [11] which may confound the results of the *in vivo* trial and 2) the limitation of studying the direct effects of feeding additives and vaccines during the challenge (e.g., competitive exclusion, toxin binding, etc.) which is mainly important in studies aimed at testing the ability of some additives in counteracting PWD. Although the ETEC challenge model has been widely used in several studies testing additives and vaccines to counteract PWD [12–17], the prevalence of pigs showing signs of infection could be low and highly variable among studies. Thus, there is a demand for optimization of the methodology and standardization of the control points in order to assure the appropriate application of the ETEC challenge model in post-weaning pigs. Therefore, this review provides an overview and evaluation regarding 1) the current piglet ETEC F4ac and F18 infection models and 2) the key clinical parameters and biomarkers of the disease which should be included in the experimental research. An additional aim of the present review was to improve the effectiveness of the protocols based on the challenge model with ETEC in order to comply with the Replacement, Reduction and Refinement (3Rs) principles, especially the Reduction and Refinement approaches as recently defined by Clark [18].

## Literature search

A literature search was conducted using PubMed, Google Scholar, Web of Science, and Scopus. The main aim of the literature research was the evaluation of ETEC F4 and F18 challenge studies in weaned piglets. Research articles in scientific journals, which were published from 1997 to January 2019 were primarily considered for data extraction for both the ETEC F4 and ETEC F18 challenge models. The following search terms in different combinations were applied to identify acceptable articles: *Escherichia coli*; ETEC F4 (and ETEC K88, according to the previous classification), ETEC F18 (and ETEC F107, 2134P and 8813, according to the previous classification); fecal score; post-weaning diarrhea and pig/porcine/piglet. Furthermore, published research studies based only on *in vitro* experiments were excluded from the studies considered.

## F4 and F18 ETEC and their putative receptors in piglets

Enterotoxigenic *Escherichia coli* strains are characterized by two types of virulence factors: 1) adhesins which allow their binding to and the colonization of the intestinal epithelium and 2) enterotoxins causing fluid secretion. The adhesins are expressed in the ETEC fimbriae, and differ between ETEC F4 and ETEC F18. Detailed information regarding fimbrial structure has been reported by Dubreuil et al. [19]. In addition, a non-fimbrial adhesin referred to as the adhesin involved in diffuse adherence (AIDA) has been recognized in ETEC strains isolated from weaned piglets with PWD [20, 21]; however, its role in PWD has still to be elucidated [22].

Once the ETEC have adhered and colonized the small intestine, they can produce enterotoxin(s) leading to diarrhea. Both ETEC F4 and F18 are recognized as producing two classes of enterotoxins, heat labile (LT) enterotoxins and heat stable (STa, STb and enteroaggregative heat-stable toxin 1 [EAST1]) enterotoxins causing electrolyte and net fluid losses [23, 24].

Currently, three serological variances of F4 have been described, namely F4ab, F4ac and F4ad, and of these, the F4ac variant has been recognized as the most prevalent in piglets [24]. Despite the differences in the antigenic classification of F4 serological variances, a common major fimbria sub-unit FaeG has been recognized as the F4 adhesin [25].

Many putative receptors have been identified for ETEC F4 adhesion showing various chemical natures and various molecular weights, as has been reported in other reviews [19, 26, 27]. Focusing on F4ac, one of the widely accepted putative receptors is constituted by two intestinal mucin-type sialoglycoproteins (IMTGP-1 and IMTGP-2) [28] linked by galactose [29]. However, these intestinal mucin-type glycoproteins have not been recognized as being responsible for transcytosis and for inducing a sufficient immune response. Thus, aminopeptidase N (APN) has been recognized as an F4 receptor (F4R) involved in the endocytosis of ETEC F4, even if it is not restricted to F4 but is also known as a receptor for some coronaviruses [30, 31]. Furthermore, a number of additional putative receptors with a glycosphingolipid nature, such as lactosylceramide, gangliotriaosylceramide, gangliotetraosylceramide, globotriaosylceramide, lactotetraosylceramide, and lactotetraosylceramide have been proposed [29, 32, 33].

Regarding ETEC F18, to date, two antigenic variants have been identified: F18ab (previously known as F107) and F18ac (previously known as 2134P and 8813) [34]. The majority of ETEC F18 strains are able to produce heat-stable enterotoxins including STa and STb [35] while the ability to produce the Shiga toxin has been more associated with F18ab [25, 36, 37]. In addition, ETEC F18ac and F18ab differ regarding their adhesion capacity; the ETEC F18ab displayed a weaker capacity to adhere both *in vivo* to ligated intestinal loops of weaned piglets and *in vitro* as compared to ETEC F18ac [37, 38]. The F18 ETEC adhere to the glycoproteins on the microvilli of the small intestine by means of their minor fimbrial subunit FedF [38, 39]. To date, a putative porcine enterocyte receptor for ETEC F18 (F18R) has been recognized to be the H-2 histo-blood group antigen (HBGAs) or its derivative A-2 HBGAs [40]. A detailed description of the ETEC F4 and F18 pathogenesis has been reviewed by Nagy et al. and Peterson et al. [41, 42].

## Setting of the challenge model

Up to now, several protocols have been published to implement the ETEC challenge model in piglets. In fact, the ETEC challenge can be carried out orally by gastric gavage or following a natural ETEC propagation by infecting a few animals in the group. The differences in the choice and preconditioning of the piglets before ETEC inoculation have been identified and should be evaluated. In addition, the timing and dosage of the ETEC inoculation, as well as the opportunity of supplying repeated dosages of ETEC, should be taken into account.

### Animal selection

Of the studies reviewed, only a few described the pre-existing sanitary conditions of the farm from which the piglets were selected. In the study of Kyriakis et al. [43], the animals were chosen from a farm with poor environmental and management conditions, and in which the piglets already exhibited the ETEC F4 infection. Other studies, including Trevisi et al. [44] and Spitzer et al. [45], took piglets from farms in which previous cases of ETEC infection had occurred in order to increase the probability of having ETEC-susceptible animals. Responses to ETEC F4 and F18 infection showed high individual animal variability, which can partially be explained by the animals’ genetic mutations associated with the expression of specific receptors on the intestinal epithelium. In order to reduce this variability, the choice of animal can benefit from specific genetic markers associated with ETEC susceptibility, which could be implemented starting from the genotyping of the sows and/or followed by piglet genotyping as described in studies conducted primarily at University experimental facilities [15, 44–47]. A wide range of genetic markers has been associated with piglet resistance to ETEC F4 and F18 utilizing association studies.

For ETEC F4, single nucleotide polymorphisms (SNPs) located on Mucin4 (*MUC4*) [48], on Mucin 13 (*MUC13*) [49, 50], Mucin 20 (*MUC20*) [51], the transferrin receptor (*TFRC*) [52], tyrosine kinase non-receptor 2 (*ACK1*) [53], UDP-GlcNAc:betaGal beta-1,3-N-acetylglucosaminyltransferase 5 (*B3GNT5*) [52] genes have been proposed as genetic markers for pig ETEC resistance/susceptibility. Goetstouwer et al. [54] have recently proposed new SNPs located on the candidate region (chr13: 144810100-144993222) as new determinates for ETEC F4 susceptibility. The proposed SNPs are located on a non-coding region and may correspond to a porcine orphan gene or a trans-acting element, which make it difficult to apply those markers as screening for the *in vivo* challenge experiments. All the above-mentioned markers are considered to be candidate markers but none of them has yet been confirmed as the univocal causative gene for F4 ETEC susceptibility, although all these markers map in the same q41 region of chromosome 13. The polymorphism located in the *MUC4* gene appears to be that most studied. Genetic population studies based on *MUC4* markers have shown that genetic susceptibility to ETEC F4 varies according to the breed. A higher prevalence of *MUC4* susceptible pigs has been observed in commercial breeds, such as the Large White, Landrace and Ukrainian breed pig lines while a lower frequency for the susceptible allele has been reported in local breeds [55, 56]. Genetically susceptible pigs showed higher diarrhea incidence and greater numbers of fecal ETEC shedding than genetically resistant animals; conversely, the phenotypic expression of F4 receptors in the intestinal brush borders displayed a large variability [57]. Based on the *in vitro* adhesion test, 30.2% of the *MUC4* genetically resistant animals showed specific receptors for F4ac and F4ab adhesion on the intestinal villi [58]. Thus, it is believed that F4 susceptibility involves gene epistasis. Furthermore, this could also be due to the limitation of the *MUC4* genotype as the causative gene for ETEC F4 susceptibility. However, since genetically F4 susceptible animals (*MUC4*^*GG*^ and *MUC4*^*CG*^) showed a complete phenotypic correspondence to their response after ETEC F4 inoculation, the choice of susceptible animals based on pig genotyping may contribute to reducing individual variability in response to ETEC F4 inoculation [57]. To overcome this lack of association between *MUC4* genotypes and ETEC F4 susceptibility, the new markers proposed by Goetstouwers et al. [54] should be studied more in depth. In fact, since Goetstouwers’ markers map on a non-coding region, no protocols in addition to an Illumina chip or a next-generation sequencing (NGS) technique are available for pig genotyping. Therefore, additional studies are necessary for developing and standardizing a fast and cheap laboratory method for pig genotyping of the markers detected by Goetstouwers [54] in order to improve pig selection for an ETEC F4 challenge model.

Regarding pig resistance to ETEC F18 infection, two main SNPs localized on alpha (1,2)-fucosyltransferase (*FUT1*) [59–61] and bactericidal/permeability-increasing protein (*BPI*) [62] genes, respectively, have been proposed. Greater consensus has been attained for the SNP located on *FUT1*. Data regarding the distribution of these genetic markers in pig populations are still scarce. However, Syrovnev [56] observed a high prevalence of susceptible genotypes in Ukrainian meat bread pigs and Bao et al. [63] showed that, for the most part, the Duroc and Pietrain breeds presented the *FUT1* resistant (*FUT1*^AA^) genotype while wild boar and other Chinese pig breeds only presented the susceptible genotypes (*FUT1*^AG^ and *FUT1*^GG^). In addition, the authors observed less scientific research regarding the study of genetic influence for ETEC F18 susceptibility than for ETEC F4 as compared to the literature research in the present paper. This may be due to the fact that less attention has been paid to F18 ETEC infection as compared to ETEC F4 infection, except for countries such as Denmark, in which breeding programs already selected for F4 pig resistance have resulted in a reduction of F4-susceptible pigs from the Danish swine population.

In the present literature review, it was observed that few *in vivo* ETEC infection studies included the selection of piglets based on genetic markers associated with ETEC susceptibility (Table 1).

**Table 1:** List of ETEC F4 and F18 challenge trials including animal choices for susceptibility, their relative model settings and the observed infection indicators included

*N* ^a^	Genetics variants^b^	No. of pigs per group	Weaning	Conditioning	ETEC^c^	Challenge dose	Challenge timing^d^	Control group^e^	Indicators for challenge effectiveness	End of the trial	Ref^f^
1	*MUC4* −/− +/− and +/+	8	d28	No	F4ac	10^9^ CFU/mL	3 dpw	NA	NA	7 dpi	[67]
2	*MUC4* −/−	8	d21	No	F4ac	10 mL of ETEC culture 10^9^ CFU/mL	8 dpw	Neg	Diarrhea; RT^i^	7 dpi	[70]
3	*MUC4* −/−	6	d21	No	F4ac	10 mL of ETEC culture 10^9^ CFU/mL	8 dpw	Neg	Diarrhea; RT^i^	7 dpi	[69]
4	*MUC4* −/−	8	d21	No	F4ac	1 × 10^10^ CFU/mL	8 dpw	Neg	Abundance of *E.coli* in feces	7 dpi	[68]
5	*MUC4* +/+	10	d24	No	F4ac	5 mL of suspension containing 10^8^ CFU/mL	7 dpw	NA	Diarrhea; fecal ETEC plate counts; anti-F4 IgA and IgM in blood	14 dpi	[12]
6	*MUC4* +/+	5	d24	No	F4ac	5 mL of suspension containing 10^8^ CFU/mL	7 dpw	NA	Diarrhea; *In vitro* adhesion test	24 h pi	[44]
7	*MUC4* +/+	10 or 20	d17–18	Yes	F4ac	1.5 × 10^9^ CFU/mL or 1.7 × 10^9^ CFU/mL or 9.8 × 10^8^ CFU/mL	3 or 7 or 21 dpw	NA	Diarrhea; gut content consistency; ETEC F4 fecal shedding; serum and intestinal anti-F4 IgM, IgA and IgG	3 or 5 dpi	[14]
8	*MUC4*+/+	8	d42	Yes	F4ac	1 × 10^8^ CFU in 20 mL NaCl	From 1 to 3 dpw	Neg	Diarroea; fecal ETEC plate counts, IgA and IgM in blood	10 dpw	[64]
9	*MUC4*+/+	6	d28	Yes	F4ac	1 × 10^8^ CFU in 20 mL NaCl	From 1 to 2	Neg	Diarroea; fecal ETEC plate counts	12 dpw	[65]
10	van Haeringen laboratory *MUC4*+/+.	^g^Exp1 = 3; ^g^Exp2 = 6	d28	Yes	F4ac	1 × 10^9^ CFU/mL in 5 mL of 9% NaCl + 10 mL of 10% NaHCO_3_	2 dpw	Exp1: Neg; Exp2: NA	^g^Exp1 and Exp2: Diarrhea and fecal DM; fecal haemolic *E. coli*;	19 dpi	[91]
11	van Haeringen laboratory *MUC4*+/+	8–10	d30–32	Yes	F4ac	5 × 10^7^ CFU in 20 mL NaCl	From 1 to 2 dpw	Neg	Diarroea; fecal ETEC plate counts	10 dpw	[15]
12	*MUC13* +/+	^g^Exp1 = 16; ^g^Exp2 = 18	d24	No	F4ac	5 mL of 10^8^ CFU/ mL	4 dpw	Neg	^g^Exp1: Diarrhea; Exp2: Diarrhea; fecal ETEC quantification	13 dpi	[46]
13	*MUC4* +/+; *FUT1* +/+	F4 = 20; F18 = 18	d28	Yes	F4 and F18ac	F18ac: 10^11^ CFU/mL; F4 10^11^ CFU/mL for 2 days	7 dpw	Neg	Diarrheal; ETEC F18 and F4 fecal shedding; F18 and F4 specific antibodies in blood; *in vitro* adhesion test	20 dpi	[72]
14	Exp1:*FUT1* +/+ and *MUC4* −/− or *MUC4* +/+; Exp2: *FUT1* +/+ and *MUC4* +/+	^g^Exp1 = 12; ^g^Exp2 = 10	d16–18	No	^g^Exp1:F18; ^g^Exp2:F4	Exp1 = 1 × 10^10^ CFU/mL or 5 × 10^10^ CFU/mL; Exp2 = 1.3 × 10^9^ CFU/mL	7 or 21 d post vaccination	Neg	Diarrhea; ETEC F18 and F4 fecal shedding; Serum anti-F4 and anti-F18 IgM and IgA;	Exp1 = 7 dpi; Exp2 = 4 dpi	[66]
15	*MUC4* +/+ and *FUT1* +/+	6	NA	No	F18ab	10 mL of 10^11^ CFU/ mL	62 dpw	NA	Fecal ETEC F18; serum IgA; *in vitro* adhesion test	7 dpi	[73]
16	*FUT1* +/+	6	^g^Exp1: d25; ^g^Exp2: d30	Yes	^g^Exp1: F18ab; ^g^Exp2:F18ac	^g^Exp1: 5 mL of 6.7 log CFU; ^g^Exp2: 5 ml of 6.7 log CFU	Exp1: 11 dpw; Exp2: 13 dpw + 30 dpw the 2° challenge	NA	ETEC F18 fecal count; serum F18 IgA	8 dpi	[113]
17	*FUT1* +/+	^g^Exp1 = 4; ^g^Exp2 = 3	d28	Yes	F18ab	10 mL of 10^11^ CFU/ mL	at weaning	NA	Diarrhea; F18-specific antibodies in blood; *in vitro* adhesion test	8 dpi	[47]
18	*FUT1* +/+	9	Not pertinent^h^	No	F18	1 mL of 7.5 × 10^10^ CFU/ mL	from days 1–8 of life	Neg	Diarrhea; *E. coli* aboundance in intestinal content	8 dpi	[75]
19	*FUT1* +/+	10	Not pertinent^h^	No	F18	1 mL of 10^10^ CFU/ mL	from days 1–4 of life	Neg	Diarrhea	5 dpi	[76]
20	*FUT1* +/+	^g^Exp1 = 6; ^g^Exp2 = 5	^g^Exp1 = d28; ^g^Exp2 = d21		F18	10 mL of 10^11^ CFU/ mL	^g^Exp1: 22 dpw; ^g^Exp2: 7 dpw	Exp1: Neg; Exp2: NA	^g^Exp1and Exp 2: Fecal excretion of ETEC F18; F18- and FedF-specific IgM, IgA and IgG response in blood and saliva; *in vitro* adhesion test	Exp1:18 dpi; Exp2:21 dpi	[74]
21	*FUT1* +/+	18	d28–34	No	F18	50 mL of 3 × 10^8^ CFU/ mL	From 3 to 6 dpw	NA	Diarrhea; *E. coli* and Hemolytic bacteria count on feces; Fecal DM; plasma IgA and IgG	Not reported	[17]

^a^*N* Study number

^b^Genetics variants: *MUC4*:Mucine4; *MUC13*:Mucine13; *FUT1*: Fucosyltransferase 1. +/+ stands for the genotypes associated with the susceptibility to the respective ETEC; −/−: the genotypes associated with the resistance

^c^*ETEC* Enterotoxigenic *Escherichia coli*

^d^Challenge timing: it was reported as days post-weaning (dpw)

^e^Control group: *Neg* Negative control group (not infected), *Pos* Positive control group (group treated with antibiotic)

^f^*Ref.* Reference

^g^*Exp* Experiment

^h^Not pertinent: performed in newborn cesarean-delivered pigs

^i^*RT* Rectal temperature

*Additional abbreviations*: *D* day, *CFU* Colony forming units, *NA* Not available, *dpi* Days post-inoculum

For ETEC F4, a total of fifteen studies were found and, of these, the most frequently used genetic markers were present in the SNP located on *MUC4*, for which genotyping was applied in ten out of the fifteen studies. Pig genotyping has been applied for different purposes. In the studies of Fairbrother et al. [14], Trevisi et al. [12, 33], Sørensen et al. [64] and Sugiharto et al. [65], the pigs were genotyped for the *MUC4* genetic marker in order to choose the genetically-susceptible pigs to be included in the trial. With the same purpose, Girard et al. [46] adopted the *MUC13* genetic marker while both genetically susceptible and resistant pigs were included in the studies of Nadeau et al. [66] and Sargeant et al. [67] with the purpose of investigating the differences in kinetics and localization of the immune response for the development of an effective vaccine. On the other hand, Yang et al. [68], Zhang et al. [69] and Zhou et al. [70] decided to include genetically resistant animals (*MUC4*-negative pigs) in an *in vivo* challenge studies with a specific ETEC F4 hybrid expressing virulence factors STb, LT and Stx2e, attaching and effacing intimin (eae), translocated intimin receptor (tir), escV, and *E. coli*-secreted protein A (espA). These studies showed that ETEC strains with different virulence capacities can cause enteritis in *MUC4*-resistant piglets. Nevertheless, it is important to note that *MUC4* has been indicated as a marker for the ETEC F4ac receptor (F4acR), and that this strain is characterized only by STb, LT and EAST1 enterotoxins [71]; thus it is possible that different F4 strains can induce infection in more complex mechanisms which are yet to be elucidated.

To date, nine studies which included pig choice based on the genetic marker for ETEC F18 resistance have been reported (Table 1). Genetically susceptible piglets (for the *FUT1* marker) have been included in studies to determine the kinetic dynamics of immune responses [72], plasma metabolites and immune response [17] to test immunization strategies, including vaccines [66, 73, 74], or to test additives to protect against infection [15, 75, 76]. Furthermore, three out of nine studies were carried out on newborn piglets in order to propose the ETEC F18 challenge as a model for humans [75–77]. Although studies regarding infectious challenge models based on *FUT1* are scarce, more recent studies carried out on healthy piglets have pointed out that the *FUT1* genotypes can influence the intestinal microbial profile [78, 79], the expression of the intestinal genes [80], intestinal mucosa protein glycosylation [81], piglet blood metabolomics [78, 79], and piglet growth performance [82] under normal healthy conditions. Thus, implementation of the *FUT1* marker in future ETEC F18 challenge studies would be beneficial in order to reduce the variability due to the genetic effect in the response data.

In addition to piglet screening for pathogen susceptibility, the pathogen-specific immunization of piglets and sows should be evaluated. In fact, beyond the passive immunity derived from sow’s milk which can affect the piglet’s responsiveness to ETEC immediately after weaning, it has been shown that maternal immunity can persist in piglet blood and can induce a systemic immune response in the piglets [83], resulting in a less efficient piglet response to the ETEC challenge. Therefore, in studies where feeding strategies with the aim of contracting the ETEC infection, selecting piglets from sows not specifically immunized for ETEC and not infected with the pathogen previously has been recommended. For studies where vaccine strategies are tested, the passage of maternal immunization should be considered for a correct interpretation of the results, as suggested by Nguyen et al. [83].

### Animal pre-conditioning

Preconditioning procedures should be carried out to contain the within variability of the piglets’ response to the ETEC challenge on the basis of their physiological status before the infection. Among preconditioning procedures, pigs can initially be treated with antibiotics, including colistin (50/60 mg per pig) [45, 84–86] or florfenicol (2 mL per pig) [87], in order to keep the animals in a healthy condition before ETEC inoculation or to contrast the effects of the weaning transition. However, this practice poses some risks; in fact, the prolonged administration of antibiotics can reduce the gut microbial variability, compromise the gut eubiosis and impair animal health [88]. Therefore, the potential antibiotic administration should usually be restricted to narrow-spectrum antibiotics and only for the first 3–4 d post-weaning [13, 89].

Furthermore, an additional practice for increasing and standardizing the piglets’ response to ETEC inoculation consists of having the animals fast for 3 h before infection and then subsequently administering 62 mL of a 1.4% NaHCO_3_-solution in order to neutralize the gastric pH before ETEC inoculation [90]. This procedure has been applied mainly in studies aiming to test immunization strategies [72, 73, 91].

### Control groups

Overall, twenty-six out of forty-eight studies included an additional negative control group (Tables 1 and 2). Including a negative control group is recommended for *in vivo* experiments and could be mandatory in experiments testing drugs [92]. This could represent a critical aspect in the case that insufficient parameters of proven infection are included in the study. However, if a good health status of piglets is guaranteed before the ETEC inoculation and a positive control group is included (i.e. an antibiotic group), the negative control group could be redundant [93]. On the other hand, if it is hypothesized that a given feed additive or a nutritional treatment is influencing the progression of the PWD via immunological mechanisms it is recommended to include a non-challenged group with the same dietary treatment.

**Table 2:** List of ETEC F4 and F18 challenge trials relative to the model setting and the infection indicators observed

*N* ^a^	No. of pigs per group	Weaning	Conditioning	ETEC^b^	Challenge dose	Challenge timing^c^	Control group^d^	Indicators for challenge effectiveness	End of the trial	Ref.^e^
1	64	d28	No	F4ac in farm	NA	Already present at weaning	NA	Fecal score; post-mortem examinations	28 dpw	[43]
2	24	d25	Yes	F4ac	10 mL of 8.1 log_10_ CFU/mL	4 dpw	NA	Fecal score; counting of haemolitic bacteria; *in vitro* adhesion test	26 dpi	[86]
3	6	d18	No	F4ac	1 × 10^9^ CFU/mL	1 dpw	Neg. and pos	Fecal score; fecal coliforms count	6 dpi	[101]
4	8	d28	No	F4 + O149	2 × 10^10^ CFU/mL	From 1 to 2 dpw	NA	Diarrhea, ETEC shedding in feces	11 dpi	[100]
5	24	d28	No	F4+ O149	6, 8 and 10 mL of 3.44 × 10^8^ CFU/mL	From 5 to 7 dpw	NA	Diarrhea; ETEC fecal shedding	7 dpi	[112]
6	8	d28	No	F4	3 mL of 3 × 10^10^ CFU/mL	7 dpw	Neg. group	Diarrhea; ETEC shedding in feces; Post-mortem examination	7 dpi	[104]
7	6	d 25	No	F4	% mL of 5 × 10^8^ CFU/mL	At weaning	NA	Diarrhea; ETEC shedding in feces	7 dpi	[99]
8	24	d 21	No	F4	6, 8 and 10 mL of 2.16 × 10^8^ CFU/mL	From 3 to 6 dpw	Neg	Diarrhea: haemolytic *E. coli* fecal shedding	11 dpi	[103]
9	12	d21	Yes	F4ac	2 mL of 5.0 × 10^9^ CFU/mL	From 3 to 4 dpw	Neg	General condition scores of pigs behaviour; diarrhea; RT^g^; fecal fae and est-II concentrations	7 dpi	[45]
10	7	d28	No	F4	1.5 mL of 10 × 10^10^ CFU/mL	14 dpw	NA	Diarrhea; *E. coli* in feces; blood Igs	28 dpi	[107]
11	10	d24	No	F4	1.5 mL of 10 × 10 ^8^ CFU/mL	7 dpw	Pos	Diarrhea; ETEC shedding in feces; blood total IgA and F4ac-specific IgA	14 dpi	[13]
12	5	d21	No	F4	1 mL of 1 × 10^9^ CFU/mL	7 dpw	Neg and Pos	Diarrhea	8 dpi	[105]
13	6	d21	No	F4	1 mL of 1 × 10^9^ CFU/mL	9 dpw	Neg and pos	Diarrhea	12 dpi	[115]
14	5	d28	No	F4	1 × 10^8^ CFU/mL	14 dpw	Neg	sIgA in bile	2 dpi	[108]
15	18	d17	No	F4	30 mL of an alkaline broth 10^10^ CFU/mL	7 dpw	Neg	Diarrhea	7 dpi	[106]
16	3–7	NA	Yes	F4	NA	At weaning	Neg	Diarrhea; serum Igs	11 dpi	[16]
17	5	NA	No	F4	10^9^ CFU/mL	12 dpw	Neg	*In vitro* adhesion test	24 h after infection	[102]
18	8 (28 in CO^f^)	d21	Yes	F4ac	1.5 mL suspension of 10^10^ CFU/mL	5 dpw	Neg	Diarrhea; blood specific-F4 IgA; *In vitro* adhesion test; ETEC plate counts on intestinal content	50% on 4 dpi; 50% on 18 dpi	[116]
19	24	d21	No	F4	1.5 mL suspension of 10^10^ CFU/mL	7 dpw	NA	Diarrhea; fecal *E. coli* and ETEC count; blood specific-F4 IgA; *In vitro* adhesion test	7 dpi	[140]
20	20	d21	No	F4	1.5 mL suspension of 10^10^ CFU/mL	2 dpw	NA	Diarrhea; fecal *E. coli* and ETEC count; blood specific-F4 IgA; *In vitro* adhesion test	6 dpi	[128]
21	12	d21	No	F4	1.5 mL suspension of 10^10^ CFU/mL	4 dpw	Pos	Diarrhea; ETEC F4 count in feces and intestinal content; blood specific-F4 IgA; *In vitro* adhesion test	11 dpi	[89]
22	12	d21	No	F4	1.5 mL suspension of 10^10^ CFU/mL	1 dpw	NA	Diarrhea; ETEC F4 count in feces and intestinal content; blood and saliva specific-F4 IgA; *in vitro* adhesion test	7 dpi	[127]
23	9	d17	Yes	F4	6 mL of 2 × 2 × 10^10^ CFU/mL	9 dpw	NA	Diarrhea; *E. coli* quantification in colon	7 dpi	[110]
24	12	d49	No	F4	20 mL NaCl with 10^8^ CFU/mL	From 2 to 4 dpw	Neg	Fecal DM	10 dpi	[118]
25	40	d25	No	F4	3 × 10^9^ CFU/mL	3 dpw	NS	Diarrhea; fecal *E. coli* counts;	5 dpi or 9 dpi	[117]
26	12	d20	Yes	F18ab	5 mL of 1 × 10^10^ CFU/mL	21 dpw	Neg	Diarrhea; fecal haemolitc colony; blood IgA and IgG;	30 dpi	[84]
27	18 (28 in CO^f^)	d28	No	F18ac	10^11^ CFU/mL	At weaning for 3 d	NA	Diarrhea; ETEC F18 quantification in feces and intestinal content	9 dpi;	[114]

^a^*N* Study number

^b^*ETEC* Enterotoxigenic *Escherichia coli*

^c^Challenge timing: it was reported as days post-weaning (dpw)

^d^Control group: *Neg* Negative control group (not infected), *Pos* Positive control group (group treated with antibiotic)

^e^*Ref.* Reference

^f^*CO* Control group

^g^*RT* Rectal temperature

*Additional abbreviations*: *D* day, *CFU* Colony forming units, *NA* Not available, *dpi* Days post-inoculum

### Timing of the inoculum

The timing of ETEC inoculation is an important point to consider for a successful pig challenge model.

The expression of F4R on the brush border membrane of the small intestine has been reported to be equally present at 1 week, 5 weeks and 6 months of age [94]. While contradictory results have been reported for the expression of F4R in the mucosa of the small intestine, Willemsen and de Graaf [94] observed no difference in 7-day-old and 35-day-old piglets and only rare detection of F4R in 6-month-old pigs. Conway et al. [95] reported an increase in F4R expression in from 7-day-old piglets up to 35-day-old pigs. In the first weeks of life, the increase in F4R expression in the mucosa according to increased age has also been proposed as one of the mechanisms which favor ETEC F4 infection in piglets [95].

Scarce information is available regarding the age-depended expression of F18R. The *in vitro* adhesion test on porcine intestinal villi showed the absence of F18R at birth in genetically-susceptible piglets; it then increased in 3-week-old piglets, and subsequently higher expression appeared post-weaning and was maintained until 23 weeks of age [40]. However, the results reported by Nadeau et al. [66] showed an increase in the specific immune response (F18-specific IgA) and in diarrhea severity in 18-day-old pigs, suggesting that F18R was already expressed at this age. Furthermore, a positive response to ETEC F18 inoculation has been observed in 0- to 7-day-old cesarean-delivered piglets, supporting the theory that F18R could be present in the early phase of life [77]. Additional experiments are required to draw a conclusion regarding the age-dependent presence of F18R since the divergent results obtained until now are difficult to compare due to differences in detecting the F18R as well as to differences in the experimental conditions.

Overall, the age-dependent expression of F4 and F18 receptors in the small intestine could contribute to explaining why ETEC F4 infection mainly occurs during the neonatal period and at weaning while ETEC F18 infection mainly occurs together with weaning and later in the piglet’s life during the growing period.

Furthermore, the multifactorial stress of weaning followed by a drop in passive immunity increases the risk of developing intestinal dysbiosis, and subsequent colibacillosis due to ETEC [96–98].

To take advantage of the stressful situation and intestinal dysbiosis, which characterize weaning, some authors have carried out the ETEC F4 or F18-inoculation on the day of weaning [16, 99] or one-day post-weaning [89, 100–102]. However, it should be considered that the passive immunity derived from sow milk immunoglobulins can influence the piglet’s response to the pathogen, causing reduced infection effectiveness. Therefore, the majority of studies have carried out the first ETEC challenge at from 3 or 4 d post-weaning [45, 46, 67, 86, 103] to 1-week post-weaning [12, 44, 104–106] due to the consideration that, during this time period, passive immunity decreased, and the piglets were still affected by the critical issues resulting from weaning. However, the effectiveness of the ETEC challenge probably depends on the weaning age and on the weight of the piglet. In studies in which the ETEC F4 inoculation was carried out 14 d post-weaning (dpw), no problem of passive immunity can be expected [107, 108]; however, the piglets could have acquired a higher immune competence to respond to the infection (Tables 1 and 2) [109]. It is rather difficult to assess when the immune system of the piglet is fully developed, and several factors beyond weaning age and weight probably influence this process. However, generally speaking, piglets are considered immunologically stable by 6–8 weeks of age [109].

Furthermore, the timing of the challenge can vary according to the objective of the study. The majority of the studies reviewed had the prophylactic effect of feed additives to counteract PWD as the main objective to investigate. According to this, the given feed additive should be provided some days before the ETEC inoculation, and hence, the timing of the challenge could be approximately 1-week post-weaning. A different objective has been proposed by Cilieborg et al. [75] and Andersen et al. [76] in which 1,2-fucosyllactose and *Lactobacillus paracasei* or *Pediococcus pentosaceus* in milk formulas were tested to counteract the ETEC F18 infection in new-born piglets as a model for human infants.

### Inoculation method and dosage

Enterotoxigenic *Escherichia coli* infection is commonly induced by the pathogen via oral administration. Less frequently, the infection has been induced by an intragastric inoculum of the pathogen, for the most part in studies aimed at developing vaccines (for ETEC F4 [55]; for ETEC F18 [73, 74]). Although the intragastric gavage allows the inoculum dosage to completely reach the gastrointestinal tract, it represents a painful and stressful procedure for the piglets. Therefore, in order to minimize the piglets’ pain and comply with the Refinement approach expressed in the 3R strategy [18], an oral inoculum should be preferred.

**Fig. 1:**
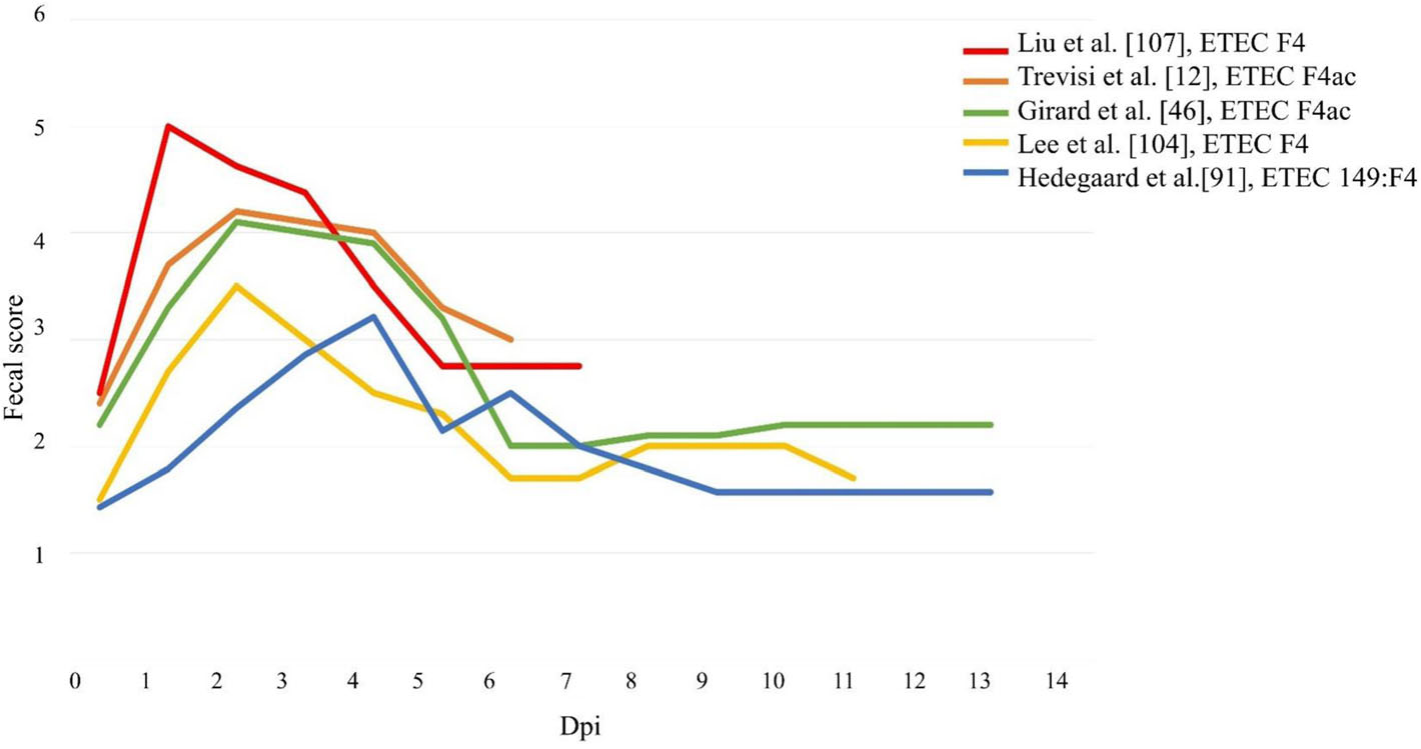
State of the fecal score consistency following enterotoxigenic *Escherichia coli* (ETEC) F4 inoculation. Data from different studies were reported on a fecal scale from 1 (dry) to 5 (watery). Liu et al. [107]: ETEC F4; Trevisi et al. [12]: ETEC F4 ac; Girard [46]: ETEC F4ac (LT+ and STb+); Lee [104]: ETEC F4; Hedegaard [91]: ETEC F4 (serotype O149:F4). Dpi: days post-inoculum

In ETEC F4 infection studies, the dosage of inoculum administered to weaned piglets varied, being approximately 10^8^ colony-forming units (CFU), i.e., 1 × 10^8^ CFU [100], 5 mL of 1 × 10^8^ CFU [12], 5 mL of 5 × 10^8^ CFU [99]. Higher dosages, 1.5 mL of 10^10^ CFU and 6 mL of 2 × 10^10^ CFU, have been administered by Trevisi et al. [13] and Molist et al. [110], respectively. Other authors induced the infection using repeated administration of the same dosage of ETEC; e.g., 1 × 10^8^ CFU, for two consecutive days [64, 65]. In some studies, increased dosages of ETEC F4 were used, i.e. piglets were challenged with 6, 8 and 10 mL of 3.44 × 10^8^ CFU/mL on days 5, 6 and 7 post-weaning [111]; with 6, 8 and 10 mL of 2.16 × 10^8^ CFU/mL for three consecutive days post-weaning [103]; with 2 mL of 5.0 × 10^9^ CFU/mL twice a day on three consecutive days post-weaning [45]. Despite the difference in the dosage used for the ETEC F4 inoculation, the first diarrhea signs were reported in all studies at approximately 24 h post-inoculum (Fig. 1). Similarly, newborns (3 d of age) challenged with 5 mL of 1 × 10^9^ CFU developed diarrhea within 6 h post-inoculum [112].

Regarding the ETEC F18 inoculation, pathogenic dosages varied from 5 mL of 10^8^ CFU/mL [113], 5 or 10 mL of 1 × 10^10^ CFU/mL [66, 84] to a higher dosage of 10 mL of a 10^11^ CFU/mL solution used by Coddens al. [47] and Verdonk et al. [72] in weaned piglets (28 and 35 d old respectively), and Tiels et al. [73] in growing pigs (at 62 d post-weaning) while three consecutive dosages of 10^11^ CFU/mL were used by Yokoyama et al. [114] in weaned piglets (28 days old). However, diarrhea has also been induced using a lower dosage of ETEC F18 inoculation, i.e. 3 × 10^8^ CFU [17].

Overall, it can be noted that for both F4 and F18 ETEC challege protocols, the dosages used are very close to the minimum dosage able to induce infection [14]. Furthermore, although the range of dosage inoculum did not vary very much among the studies, and the pigs developed diarrhea, a high variability in diarrhea severity and diarrhea incidence have been observed (see the section “Diarrhea and related indicators”). A large experimental variation in diarrhea outcome can be due to individual animal variability among the studies i.e. genetic susceptibility and animal immune competence. In addition, the natural exposure of *E. coli* from the sow and/or the environment may contribute to variation within an experiment.

## Evaluation of the challenge effectiveness

A wide range of response indicators has been proposed in ETEC challenge studies, including both clinical and behavioral parameters. Clinical signs for a complete diagnosis have recently been described by Luppi [24] while Jensen et al. [71] and Spitzer et al. [45] proposed scoring the pigs according to their general condition with a 1–4 point score where 1 = no impairment of health; 2 = mild impairment: reduced activity, atypical behavior, reduced feed intake; 3 = moderate impairment: inactivity, weakness, feed refusal and 4 = severe impairment: inappetence, dehydration and reduced body temperature. However, these parameters have been criticized. In fact, they need to be reported by the same trained person, they are time-consuming and they are not widely utilized among the studies; thus, they were not useful for the present review. Therefore, in this review, the most acceptable response indicators, which allowed determining whether the ETEC challenge was successfully carried out were identified and described. The parameters identified included clinical parameters, such as the occurrence of diarrhea, rectal temperature (RT), and stimulation of immune response or the isolation of pathogens in the feces. Among the indicators described, some were considered pathogen-specific, thereby allowing the proper association of the pig response to the inoculated ETEC strain, resulting in effective evidence of a successful challenge protocol.

### Diarrhea and related indicators

The development of the clinical disease symptom (diarrhea) and the related indices, including its frequency and severity, are the most accepted response parameters for evaluating ETEC infection. Those diarrhea indicators can be assessed using different methods, including evaluation of fecal consistency scores, fecal dry matter (DM) and days of diarrhea.

The most frequently used fecal score classification is summarized in Table 3. The most frequently used fecal score classification is based on a continuous scale of 5 levels which evaluate fecal consistency where 1 = hard and dry feces; 2 = well-formed firm feces; 3 = formed feces; 4 = pasty feces and 5 = liquid diarrhea [12, 13, 67] or conversely from 1 to 5 where 1 = watery feces and 5 = hard feces [45], and where a consistency score > 3 is defined as a clinical sign of diarrhea. Scoring can be extended to 7 levels and classified for feces consistency and color, according to the Bristol Stool Scale where a consistency score > 3 is defined as a clinical sign of diarrhea [91] or reduced to 4 levels (1 = normal feces, 2 = soft feces, 3 = mild diarrhea, and 4 = severe diarrhea [104, 111] or to 3 level [115] (Table 3).

**Table 3:**
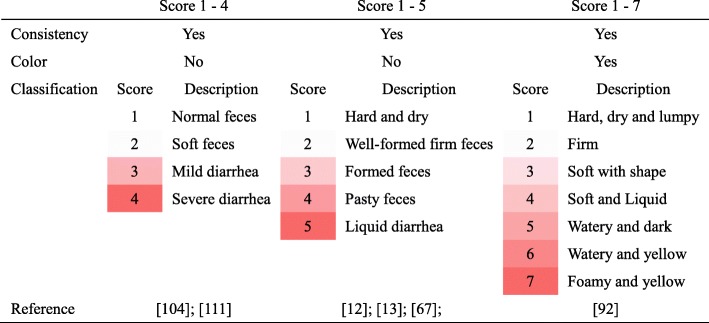
Assessment of the pigs fecal score

Color: intense red indicates the threshold for diarrhea classification and its severity

Overall, one of the most important aspects is the collection time of the fecal consistency data. Fecal score recording should start from the day before ETEC inoculation in order to check that the health status of the animals is good before inoculation, and continue daily during the first-week post-inoculation and, subsequently, every second day, preferably until the piglets recover.

The majority of the studies which carried out the ETEC F4 inoculation during the first week post-weaning reported an impairment of fecal score consistency from 24 h’ post-inoculum [12, 45, 104] (Fig. 1) while, in neonatal piglets, the impairment of the fecal score was already observed 3 or 6 h after F4 inoculation [112]. Thus, it is very important to record the fecal score consistency in the first 24–36 h after ETEC inoculation in order to identify the exact timing of the appearance of the diarrhea. Overall, the peak of the diarrhea (the worst fecal score) has been observed to be from 2 to 4 d after ETEC F4 inoculation up to a week later (Fig. 1).

Differences in the timing of the diarrhea occurrence may be due to individual variability. In fact, piglets with a higher expression of F4Rs on the intestinal brush border showed an earlier manifestation of diarrhea and the worst fecal score [13, 71, 116].

Data regarding the fecal consistency score could also be reported as diarrhea incidence defined as the percentage of piglets with diarrhea on a specific day after ETEC inoculation. Differences in diarrhea incidence were observed among the studies. Considering the positive control group of the different studies, it could be observed that ETEC F4 inoculation induced a diarrhea incidence reaching 40–50% at 3 d post-inoculum (dpi) [86], 5 dpi [117] and 7 dpi [12] while it reached 80% in the studies of Pan et al. [115] at 3 dpi. A reduction in diarrhea incidence has been observed within 11 dpi by Pieper et al. [117] and Kiers et al. [86] despite the difference in F4 ETEC dosages.

Continued monitoring of the fecal consistency score from the day of inoculation to the end of the trial allowed calculating the days with diarrhea which reflected the animal recovery.

Fecal DM is a frequently used indicator of porcine diarrhea, and it is inversely correlated with the diarrhea assessed using fecal scoring, i.e. higher fecal DM when there is less diarrhea. It is determined in samples obtained from individual pigs taken daily from day 1 before the challenge until the end of the challenge [45, 64, 91, 118]. Few studies have reported fecal DM determination in parallel with the diarrhea score, although fecal DM is not prone to subjective evaluation as with fecal scoring. In F4 inoculated piglets, fecal DM decreased from 24.7% in pre-challenge conditions to 12.9–20.4% during 1 to 3 dpi. A normal fecal DM was then recovered within 5 dpi [45].

**Fig. 2:**
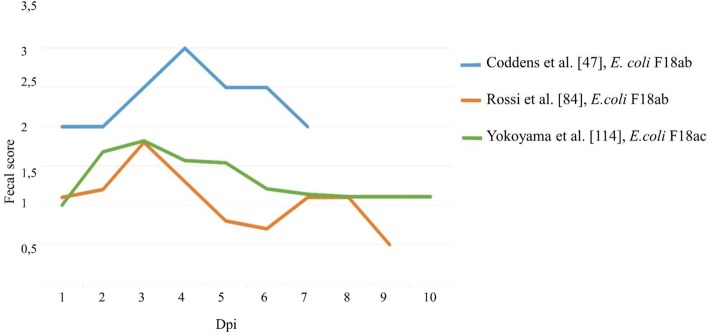
State of the fecal score consistency following the ETEC F18 inoculation. Data from different studies were reported on a fecal scale from 1 (dry) to 4 (watery). Coddens et al. [47]: *E. coli* F18ab-positive, *E. coli* strain107/86 (serotype O139:K12:H1, F18ab+, SLT-IIv+, resistant at 1 mg/ml streptomycin; Rossi et al. [84]: *E. coli* F18ab-positive, (serotype O138, VT2e+); Yokoyama et al. [114]: *E. coli* F18ac, *E. coli* strain 8199 (serotype O141ab: H4: F18ac+: STIa, STII)

Information regarding diarrhea due to F18 ETEC inoculation is scarce in comparison with that regarding F4 ETEC, and studies have shown a high variability in diarrhea response despite quite similar inoculation dosages (Fig. 2). The high variability in diarrhea response shown in Fig. 2 could be due to the serological variants of the *E.coli* used in the various studies. In fact, Coddens et al. [47] used *E.coli* serotype O139:K12:H1, Rossi et al. [84] used *E. coli* serotype O138 and Yokoyama et al. [114] *E. coli* serotype O141. A less severe diarrhea outcome was observed by Rossi et al. [84] and Yokoyama et al. [114] as compared to Coddens et al. [47]. The more severe diarrhea observed by Coddens [47] could also be due to the choice of genetically susceptible animals. On the contrary, Verdonck et al. [74] reported, in piglets genetically susceptible to ETEC F18 and treated with the same ETEC dosage and strain used by Coddens, a low diarrhea response. Measuring the fecal consistency and the fecal DM, Sugiharto et al. [17] observed that 30–40% of ETEC F18-susceptible piglets suffered from diarrhea 3–4 d post-weaning, with the first F18 inoculum provided to piglets at 1 d post-weaning, i.e. a similar trend in diarrhea development to the F4 inoculation experiments (Fig. 1). Since the genotype cannot discern the magnitude of the piglets’ susceptibility, the differences observed may be due to the different expression of F18Rs on the intestinal brush border. In fact, the comparison of F18R expression between piglets with susceptible genotypes still remains to be studied. Furthermore, differences in the occurrence of diarrhea among the studies could be due to the F18 strain used and to its virulence. For instance, Yokoyama et al. [114] adopted an ETEC F18ac strain while other authors used an ETEC F18ab strain. It is difficult to draw a conclusion regarding the timing and severity of diarrhea due to ETEC F18 inoculation with the data available; thus, additional studies are necessary to correctly describe the diarrhea manifestation as a valid criterion for assessing F18 challenge protocol.

### Rectal temperature

An additional clinical indicator for pig health status is body temperature. Body temperature is commonly assessed using RT which has been considered to be one of the best indicators of core body temperature [119]. In challenge studies, the RT is measured daily from day 1 before inoculation until 7 dpi, using an electronic thermometer [45, 104]. Pig RT ranges from 39.0–39.5 °C pre-challenge to > 40.0 °C 6 h’ post-inoculation, and it then decreases gradually. High variability has been reported for the time necessary for the rectal temperature to return to a physiological level. For an ETEC F4 challenge, the timing can vary from 24 h after inoculation [101] to 2 or 3 dpi [104], or to 5 dpi [45]. However, some concerns have been associated with RT detection. Obtaining RT could be time-consuming and is stressful for the animals, especially for sick animals. Furthermore, it may be inaccurate due to the presence of watery feces in the rectum and the animal’s movements [119, 120]; therefore, in the present survey, this measurement was reported in very few studies.

### Bacterial fecal shedding

Bacterial shedding has been widely recognized as an indicator for evaluating the host responses to infection; however, differences in bacterial species and in the timing of the analyses have been observed. The most accurate information is provided by the evaluation of ETEC F4 and F18 fecal shedding in the period from before inoculation to 3–4 dpi. This time period after inoculation is required to allow the ETEC to adhere, colonize and produce toxins in the small intestine.

Differences in the time for ETEC F4 and F18 fecal excretions post-inoculation have been reported. The peak of ETEC F4 excretion following the ETEC F4 inoculation (10^11^ CFU) is at 2 dpi (5.97 × 10^8^ F4 per gram of feces); a sudden decrease in the ETEC F4 fecal count then already occurs at 3–4 dpi [72]. Verdonck et al. reported a similar level of F4 fecal shedding [72] at 3–4 dpi using lower F4 ETEC dosages [12 (10^8^ CFU/mL), 13 (10^10^ CFU/mL)].

For ETEC F18, the peak of fecal excretion occurred 3–5 dpi (9.9 × 10^7^ F18 per gram of feces); contrary to F4 excretion, the amount decreased gradually and it resolved between 9 and 11 dpi [66, 72, 73, 113]. Hence, the intestinal colonization of ETEC F4 seemed somewhat faster than for F18. This could be explained by the different amounts of adhesin in the fimbriae of ETEC F4 and F18. The adhesion of F4 fimbriae is mediated by the major subunit FaeG while, for F18 fimbriae, the adhesin is expressed by the minor subunit FedF, resulting in a lower ETEC F18 ability to adhere to the specific receptors on intestinal enterocytes, causing a lower immune response and slower pathogen excretion [39, 72, 74]. In addition, small differences in fecal shedding between the two F18ac and F18ab strains can be observed. In fact, the F18ac strain shows a faster reduction in fecal excretion than the F18ab strain [113].

Overall, the authors observed that the evaluation of F4 and/or F18 fecal shedding was carried out in only seventeen out of forty-five studies (Tables 1 and 2). Regrettably, in the authors’ opinion, this was not adequate considering the important information that this analysis obtained. Specific protocols for ETEC F4 and F18 isolation from feces and their characterization can be found in Nadeau et al., Verdonck et al. and Loos et al. [23, 66, 72]. Briefly, ETEC F4 and F18 isolation consists of diluting 10 g of feces 10-fold in peptone water and subsequent anaerobic incubation of the dilutions selected into 5% bovine blood agar plates containing 50 μg/mL of nalidixic acid for 24 h at 37 °C. In addition to the fecal counting, the ETEC colony should be serotyped in order to verify the strain [121]. Furthermore, assessing and quantifying the pathogenic enterotoxins may be an even more precise estimate for controlling the efficacy of the ETEC challenge model since the excreted ETEC toxins indicate the level of infection. The LT, STa and STb enterotoxins can be assessed using an enzyme-linked immunosorbent assay (ELISA), a competitive enzyme immunoassay (EIA), by immunoblotting using a specific monoclonal antibody [23] or using a quantitative polymerase chain reaction (qPCR). Specific primers and conditions to detect ETEC virulence genes using PCR can be found in Byun et al. and Khac et al. [122, 123]. Furthermore, the precise detection and quantification of the enterotoxins of the ETEC strains inoculated will permit defining standard virulence ETEC strains for pig challenge models, resulting in a reduction of strain variability effects.

Other studies have provided information only on total *E. coli* fecal shedding [101, 107, 117] or measuring the CFU at the level of the colon [110]. Since *E. coli* is considered an ubiquitary bacterium, its total increase cannot be directly associated with the increase in the pathogenic strain used for the challenge; therefore, the total increase in *E. coli* is not considered a precise indicator for claiming the success of the challenge protocol.

### Immunoglobulins

Immunoglobulins (Igs) are crucial for defending organisms from pathogens and are also recognized as key players for clinical, diagnostic and biotechnological applications. Therefore, Igs have been exploited as the main indicators of ETEC infection and their quantification in challenge experiments have generally been carried out using blood serum and saliva, intestinal mucosal samples or bile. Among Igs, IgG and IgM are partially ineffective for the mucosal surface while IgA contributes to the host mucosal defense since it improves the organism resistance to bacterial proteolytic enzymes and can bind antigens, preventing pathogens colonization [124]. For this reason, quantifying the secretory IgA (SIgA) is recommended and, on infection, its concentration should be higher in mucosal and/or bile samples of ETEC in infected piglets than in non-infected piglets, at least at the peak of the infection [108, 125]. However, since slaughter of the experimental piglet is necessary to obtain this information, it is not an option and, therefore, the quantification of plasma or serum IgA is carried out [17, 73] and, in parallel with hematological parameters, the IgA quantification in plasma or serum allows following up the infectious response to the ETEC challenge as demonstrated by Sugiharto et al. [17] and Rossi et al. [84]. In addition to IgA, quantification of blood IgG and IgM could allow obtaining a more accurate description of animal history regarding previous ETEC infection or regarding immunological competence derived from the mother.

In order to obtain the information most targeted to the response against ETEC F4 and F18, the quantification of pathogen-specific Igs has been applied in several studies [12, 72, 116, 126–128]. In fact, as observed by Trevisi et al. [12] the trend of total serum IgA did not reflect the trend of F4-specific IgA; thus, the analysis of total IgA rather than specific IgA could mask interesting results regarding the specific response of the piglets to the infection. The different response between total or pathogen-specific IgA could be due to the fact that total IgA production can be stimulated by the bystander activation of B cells caused, for instance, by the LPS. This bystander stimulation improved B cell mitosis and induced a polyclonal response, increasing the production of a non-specific antibody in a T-cell dependent or independent manner [129].

It should be noted that neither the ELISA kit nor F4 and F18 specific antigens are commercially available. However, the protocols for determining specific ETEC F4 and F18 have been published [72, 126]. These protocols involve the collection of F4 and F18 fimbriae to be prepared for analysis of the specific F4/F18 fimbrial antigens in a blood sample.

**Fig. 3:**
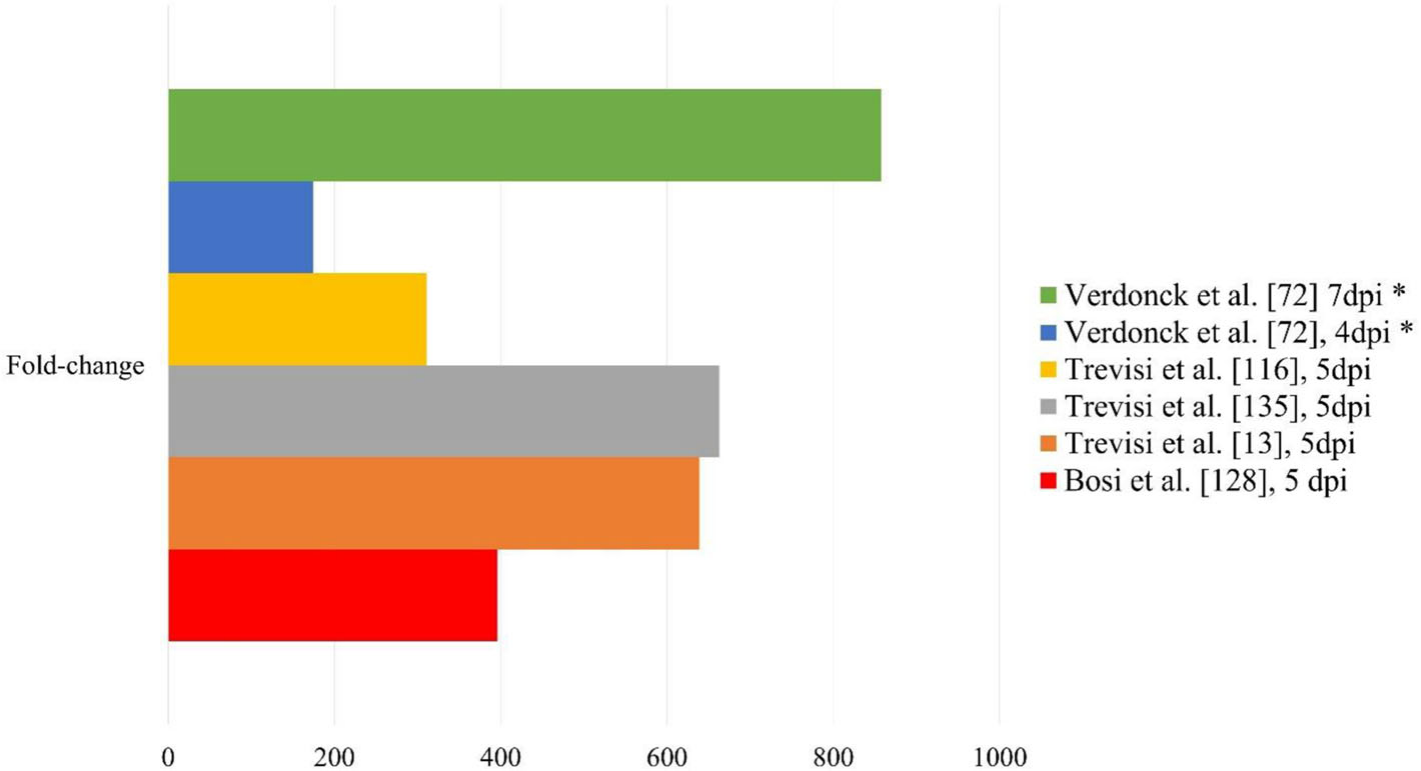
Increment of serum F4-specific immunoglobulin A (IgA) in piglets after enterotoxigenic *Escherichia coli* (ETEC) F4ac inoculation. Bars represent the fold change of F4-specific IgA in serum between the pre-challenge and the post-challenge period. * data were transformed from log_2_ values. Dpi: days post-inoculation

Differences in the immune response to ETEC F4 and F18 inoculation can be observed. The synthesis of F4-specific IgA is faster and more intense than F18-specific IgA, which can be ascribed to the higher ability of ETEC F4 to adhere on the brush border compared to ETEC F18 [72]. Specifically, the serum F4-specific IgA increased from 4 to 7 dpi, and reached log_2_ 6 titers [72] and its level stayed at this high level until 14–18 dpi [12, 13]. Several studies have observed that F4-specific IgA titers increased from 310% to 662% in the period from the pre-challenge to 4–5 dpi and reached an increase of 857% at 7 dpi (Fig. 3). Serum F18-specific IgA increased at 11 dpi and reached a maximum level at 21 dpi when its amount was reported as log_2_ 4 [72].

The serum level of specific IgA can be affected by piglet priming and by the individual amount of F4/18Rs on the brush border [89, 126].

Similar to the plasma or serum concentration of IgA, blood IgM and IgG displayed differences in timing and quantification between F4 and F18 ETEC inoculation. F4-specific IgGs in the blood started to increase at 4 dpi and achieved a plateau at 7 dpi while the F18-specific IgGs increased only after 11 dpi and reached their maximum level at 25 dpi. The F4-specific IgMs started to increase at 4 dpi and had their maximum level at 7 dpi while the F18-specific IgMs only slightly increased until 7 dpi and then decreased from 15 dpi [72].

In addition to blood serum Ig qualification, some studies developed protocols for Ig quantification in saliva and feces [84, 89, 130]. The application of non-invasive markers in an ETEC challenge study can be of notable interest to promote the refinement approach *in vivo* studies. Fecal immunoglobulin quantification has frequently been used in humans to assess intestinal permeability, intestinal epithelial barrier functionality and bacterial translocation [131]. In pigs, fecal immunoglobulins have only been scarcely investigated. In the study of Rossi et al. [89], the quantification of fecal IgA coupled with health parameters allowed assessing the piglets’ response to ETEC inoculation after vaccinations. The quantity of fecal IgA is influenced by age and by passive immunity received from the sow [130, 132]; thus, these factors need to be taken into account in longitudinal studies which use fecal IgA as an immunological marker. In addition, fecal IgA can vary according to host-microbiota interaction [133]; therefore, commensal bacteria other than the inoculated ETEC can affect the fecal IgA titer. To overcome this inaccuracy, specific fecal F4 and F18 IgA should be analyzed in ETEC challenge studies, as proposed for porcine epidemic diarrhea virus infection [134].

Saliva sampling is easy to carry out and is stress-free; however, very little information has been reported regarding Ig salivary kinetics after ETEC inoculation. The existing information is limited to IgA class and to studies using the F4 challenge model. With respect to blood F4-specific IgA, a lower level of F4-specific IgA is reported in the saliva [135]. Its level increases after the challenge up to 7 dpi [128]; however, a description of their kinetics over time is lacking. Contrary to the differences in blood F4-specific IgA between susceptible and resistant piglets, no genetic difference in F4-specific IgA is observed in saliva [136]. Some authors have suggested that the lack of difference in saliva IgA between susceptible and resistant piglets could be due to a local mechanism of the immune response from salivary glands or to sampling issues [137].

### Expression of the ETEC-specific receptor in the intestinal mucosa

The genotyping for the different markers associated with the ETEC susceptibility reported in the previous paragraph increased the likelihood of identifying ETEC F4- and F18-susceptible piglets. However, the phenotypic expression of the receptors, especially the F4R, has a large variability, and is believed to involve gene epistasis [58]. Therefore, to confirm piglet ETEC susceptibility, it is necessary to assess the expression of F4/F18 receptors on the intestinal brush border. Protocols to evaluate the presence of ETEC receptors consist of a post-mortem *in vitro* adhesion test which has been developed for both ETEC F4 and F18. This *in vitro* test consists of counting the number of ETEC F4 or F18 adhering bacteria on the brush border of the jejunum villi. Detailed protocols are explained by Van den Broeck et al. [126] for ETEC F4 adhesion, and by Verdonck et al. [74] and Yokoyama et al. [114] for the ETEC F18 adhesion. As an alternative method, an *ex vivo* approach has been proposed by Sugiharto et al. [138] which consists of an intestinal organ culture (PIOC) of ETEC and subsequent ETEC plate enumeration.

Overall, the authors observed that 12 out of the 48 studies carried out a post-mortem confirmation of piglets’ susceptibility to the inoculated ETEC strain. The results obtained were used by the authors to confirm the animal’s susceptibility to ETEC (presence or absence of receptors) or to classify the animals based on their ETEC susceptibility (number of receptors per unit of villi surface [126]). In the latter case, the authors used the *in vitro* adhesion test data as an individual scoring of piglet susceptibility; the scoring was then used to classify the animals (mildly or highly susceptible) and it was added as a factor in the statistical model [116, 139]. However, no difference between homo and heterozygous susceptible genotypes to ETEC was obtained with regard to the level of intestinal adherence of ETEC measured *ex vivo* [138].

## Conclusion and perspectives

The literature review pointed out the differences in the piglets’ response to F4 and F18 inoculation, especially in terms of the intensity and the timing of the diarrhea and of the piglets’ immunological response and their pathogen fecal shedding. Additional research is necessary to assess the piglets’ response to ETEC F18 inoculation in order to define the timing and the values of the indicators for the development of the challenge model. Table 4 summarizes the main features which need to be taken into account when designing an ETEC challenge trial, including the setting of the model and the criteria which allow a correct evaluation of the challenge effectiveness. The wide individual response variability observed among piglets to the ETEC challenge can be partially controlled by proper choice (based on genetic markers) and assessment (with the analysis of ETEC receptors) of ETEC-susceptible animals. Inclusion of pathogen-specific indicators such as specific F4 and F18 Igs, ETEC F4/F18 fecal enumeration and the in vitro ETEC adhesion test would be desirable to properly justify the effect of the specific interventions when the challenge model is applied. The above are important for the optimization of the experimental design and, in this way, take into consideration the 3R approach when using the piglet challenge model, especially concerning the issues Reduction and Refinement.

**Table 4:** Main features for assessing an ETEC challenge trial, including the setting of the model and the criteria for evaluation of the challenge effectiveness

Setting of the challenge model		
Animal selection		
Genetic markers which assist in ETEC susceptibility	ETEC F4: non coding region [54]; *MUC4* [48]; *MUC13* [49, 50]	ETEC F18: *FUT1* [60]; *BPI* [62]
Sanitary status of the farm	Previous ETEC infection on the farm; Vaccine prophylaxis	
Suggested animal preconditioning	Treatment with narrow-spectrum antibiotics	
	Fasting for 3 h + 62 mL of a 1.4% NaHCO_3_-solution	
Control group	Negative control group -- > recommended if a feed intervention is predicted to influence the PWD via immunological mechanisms	
Timing of the inoculum	4–7 dpw -- > multifactorial stress of weaning followed by a drop in passive immunity	
Inoculation method and dosage	Oral administration: to comply with the Refinement approach	
	ETEC F4: 1×10^8^ CFU/mL - 1.5 mL of 10^10^ CFU/mL	ETEC F18: 5 mL of 10^8^ CFU/mL - 10 mL of a 10^11^ CFU/mL
Evaluation of the challenge effectiveness		
Diarrhea and related indicators	Fecal score: daily from a day before ETEC inoculation until the pigs recovered	
	Diarrhea incidence: peak during the first week post-ETEC inoculation	
	Days with diarrhea: daily from a day before ETEC inoculation until the pigs recovered	
	Fecal DM: daily from a day before ETEC inoculation until the pigs recovered: 12.9–20.4% during dpi 1 to 3	
Rectal temperature	39.0–39.5 °C pre-challenge to > 40.0 °C 6 h’ post-inoculation, then decreasing gradually	
Bacterial fecal shedding	Evaluation of ETEC F4 and F18 fecal shedding in the period from before inoculation and 3–4 dpi	
	ETEC F4: 2 dpi (5.97 × 10^8^ F4 per gram of feces), then decreasing suddenly	ETEC F18: 3–5 dpi (9.9 × 10^7^ F18 per gram of feces), then decreasing gradually
Immunoglobulins	Secretory IgA: mucosal and/or bile samples	
	F4-specific and F18-specific IgA from blood plasma at 5 dpi	
Expression of ETEC specific receptors in the intestinal mucosa	Post-mortem *in vitro* adhesion test	
	Intestinal organ culture (PIOC)	

*Abbreviations*: *ETEC* Enterotoxigenic *Escherichia coli*, *MUC4* Mucine 4, *MUC13* Mucine 13, *FUT1* Fucosyltransferase 1, *BPI* Bactericidal/permeability increasing protein, *PWD* Post-weaning diarrhea, *dpw* Days post-weaning, *DM* Dry matter, *dpi* Days post-inoculum, *IgA* Immunoglobulin A, *PIOC* Intestinal organ culture

## Abbreviations

ACK1: Tyrosine kinase, non-receptor, 2
B3GNT5: UDP-GlcNAc:betaGal beta-1,3-N-acetylglucosaminyltransferase 5
BPI: Bactericidal/permeability-increasing protein
CFU: Colony-forming unit
DM: Dry matter
Dpi: Days post-inoculum
*E. coli*: *Escherichia coli*
ETEC: Enterotoxigenic *Escherichia coli*
F4/18R: F4/18 receptors
FUT1: Alpha (1,2)-fucosyltransferase
Ig: Immunoglobulin
LAB: Lactic acid bacteria
MUC4/ MUC13/ MUC20: Mucin4, Mucin13, Mucin20
PIOC: Porcine intestinal organ culture
PWD: Post-weaning diarrhoea
RT: Rectal temperature
SIgA: Secretory IgA
TFRC: Transferrin receptor
